# Colonic Metastasis of Previously Undetected Invasive Lobular Carcinoma in a 68-Year-Old Woman Undergoing Regular Breast Screening

**DOI:** 10.7759/cureus.96816

**Published:** 2025-11-14

**Authors:** Giulia Capelli, Corrado Sala, Maria Grazia Sauta, Domenico Marcello Gerbasi, Valentina Crisafulli, Giuseppe Buragina, Pierpaolo Mariani, Luca Ambrogio Rampinelli

**Affiliations:** 1 Department of Surgery, ASST Bergamo Est, Seriate, ITA; 2 Department of Medicine, ASST Bergamo Est, Seriate, ITA; 3 Department of Services, ASST Bergamo Est, Seriate, ITA

**Keywords:** atypical breast cancer presentation, cdk4/6 inhibitor therapy, colonic metastasis of breast cancer, multidisciplinary approach, negative screening mammography

## Abstract

Invasive lobular carcinoma (ILC) is a breast cancer subtype known for its subtle presentation and potential for unusual metastatic spread. We present the case of a 68-year-old woman with vague abdominal pain and a positive fecal occult blood test. Colonoscopy revealed a vegetative neoplasm in the cecum with ileocecal valve stenosis. Initial histopathology, performed during endoscopy, identified an epithelial infiltrative histotype but did not allow further characterization due to limited differentiation. The tumor was staged with a contrast-enhanced thoracoabdominal CT scan, and multidisciplinary discussion led to a videolaparoscopic right hemicolectomy. Final histopathology revealed extensive infiltration consistent with ILC, luminal A subtype. Although the patient reported regular breast cancer screening with annual mammography and ultrasound, no abnormalities had been previously detected. Postoperative imaging confirmed a retroareolar breast lesion with nipple retraction and axillary lymphadenopathy. Following multidisciplinary discussion at the Breast Unit, further histologic typing was deemed unnecessary, and the patient was started on endocrine therapy combined with a CDK4/6 inhibitor. This case highlights a rare presentation of ILC and underscores the importance of maintaining clinical suspicion for metastatic breast cancer in patients with gastrointestinal lesions and inconclusive endoscopic findings.

## Introduction

Colon cancer is a major health concern in the Western world, with surgery as the primary curative treatment [1]. However, multidisciplinary teams (MDTs) play a key role in optimizing patient outcomes, ensuring thorough evaluation and treatment planning [2]. While adenocarcinoma accounts for 98% of colorectal cancers, rarer histotypes make up the remaining 2% [3]. Among these, gastrointestinal metastases from primary breast cancer are uncommon, with colonic involvement reported in 3-12% of cases [4]. Even more unusual is the presentation of colon metastasis as the first manifestation of undiagnosed breast cancer, particularly in patients undergoing regular screening. Invasive lobular carcinoma (ILC), which represents 5-15% of breast neoplasms, is characterized by diffuse infiltration rather than nodular formation, making detection challenging with standard imaging techniques such as mammography and ultrasound [5].

We present the case of a 68-year-old woman with no history of breast cancer and regular negative screenings, who developed an epithelial neoplasm of the right colon and underwent videolaparoscopic right hemicolectomy. Histopathology revealed diffuse metastatic involvement from previously undetected ILC.

Most cases reported in the literature described patients with a prior diagnosis of breast cancer, typically ILC, who developed colonic metastases several years after initial treatment, often presenting with nonspecific gastrointestinal symptoms such as abdominal pain, altered bowel habits, or bleeding [5-18]. Only one case was found of a 73-year-old woman diagnosed with colonic metastasis from lobular breast cancer without prior evidence of a primary tumor [19].

This case highlights the need to consider breast metastasis as a rare but possible cause of colonic epithelial neoplasia, even in patients with negative screening tests. Diagnosis can be challenging and may require surgical excision for histologic confirmation.

## Case presentation

**Figure 1:**
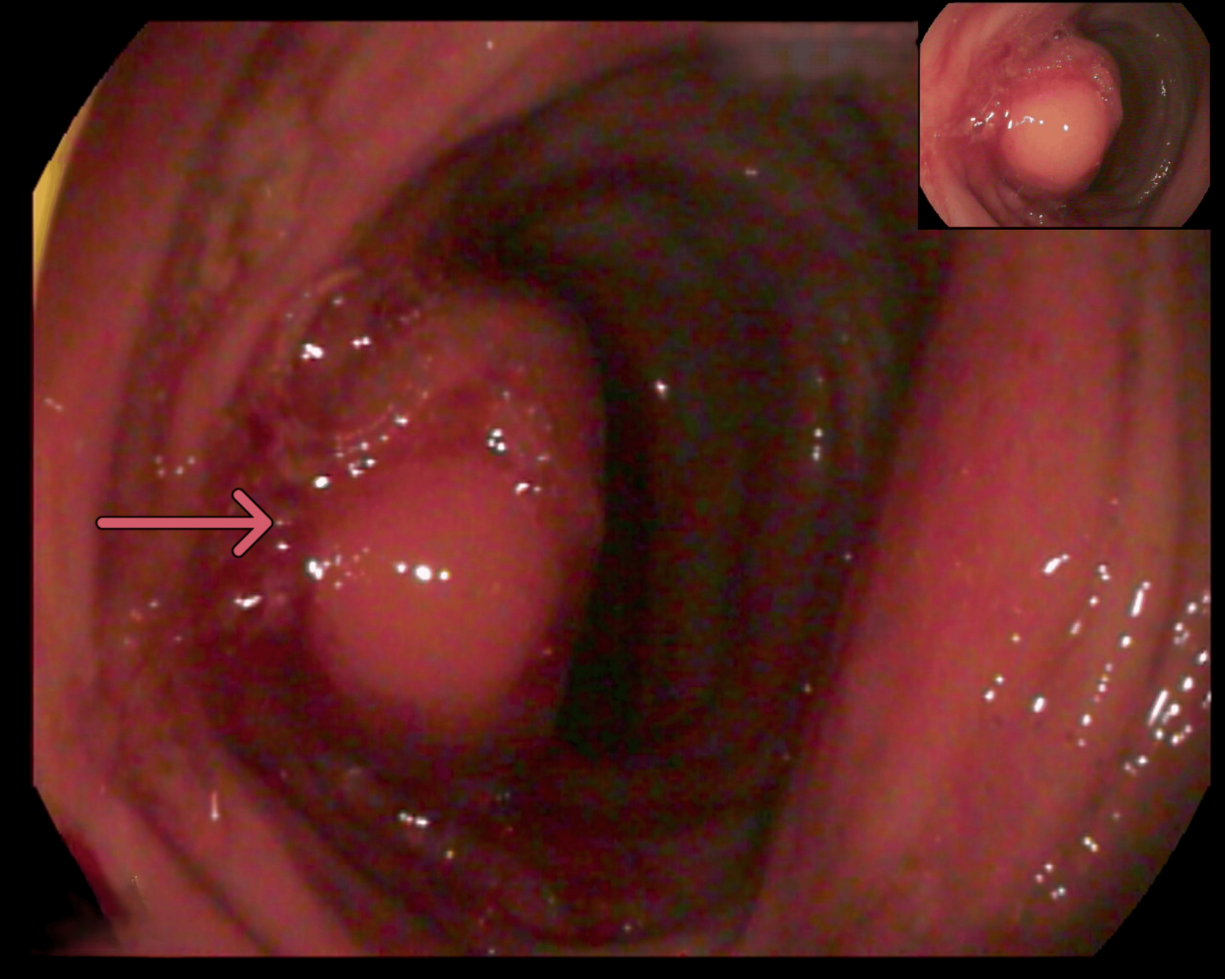
Endoscopic findings at diagnosis. Colonoscopy image showing a vegetative neoplasm in the cecum causing stenosis of the ileocecal valve. An arrow indicates the area of interest at the site of the obstructive lesion.

A 68-year-old woman with obesity, hypertension, and non-insulin-dependent diabetes mellitus presented with vague abdominal pain and a positive fecal occult blood test. She had no history of cancer, reported no rectal bleeding or changes in bowel habits, and participated in regular screening programs. In her region, colonoscopy is offered only following a positive fecal occult blood test; she had not undergone colonoscopy in the preceding 10 years. She also underwent annual breast ultrasound and mammography on her own initiative; her most recent mammogram, performed nine months earlier, showed microcalcifications in the upper outer quadrant of the left breast and a small nodular opacity in the right breast, both unchanged from prior exams. A follow-up ultrasound was unremarkable. Colonoscopy revealed a vegetative cecal neoplasm causing stenosis of the ileocecal valve, along with a polypoid lesion in the right colon (Figure 1).

The polypoid lesion was removed and identified as a low-grade adenoma, while the cecal neoplasm was classified as an epithelial infiltrative histotype without possibility for further characterization.

A thoracic and abdominal CT with intravenous contrast confirmed a cecal mass with enlarged regional lymph nodes but no distant lesions; tumor markers (CEA, Ca 19-9) were negative. As previously noted, the pathologist indicated that no further diagnostic information could be obtained from the available tissue. In this context, and in the absence of actionable pathology, the multidisciplinary team prioritized therapeutic decision-making. Videolaparoscopic right hemicolectomy was therefore recommended.

**Figure 2:**
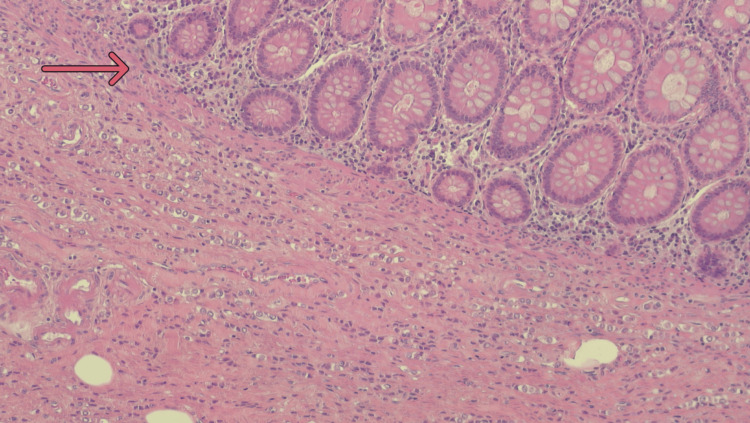
Ematossilin-eosin (EE) staining of the colon specimen. Ematossilin-eosin (EE) staining of the colon specimen at 10x magnification showing massive extensive spread in the muscular layer with relative sparing of the mucosal layer. The arrow indicates the transition zone between the mucosa (relatively spared) and the muscular layer (site of extensive metastasis).

**Figure 3:**
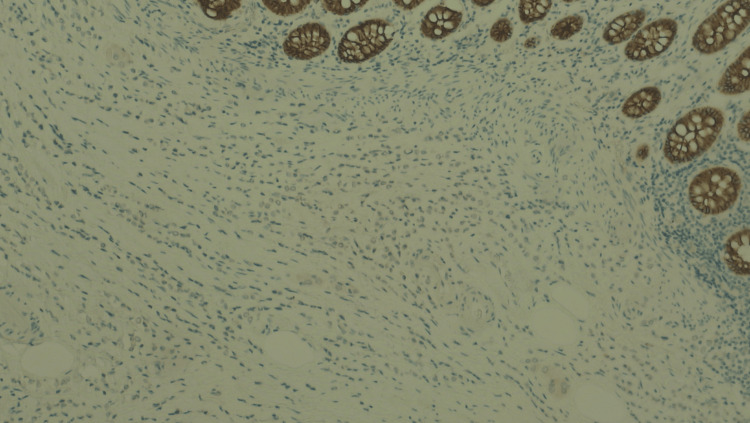
Immunostaining of the colon specimen: E-cadherin. Immunostaining for E-cadherin at 10× magnification. The tumor cells are negative for E-cadherin.

**Figure 4:**
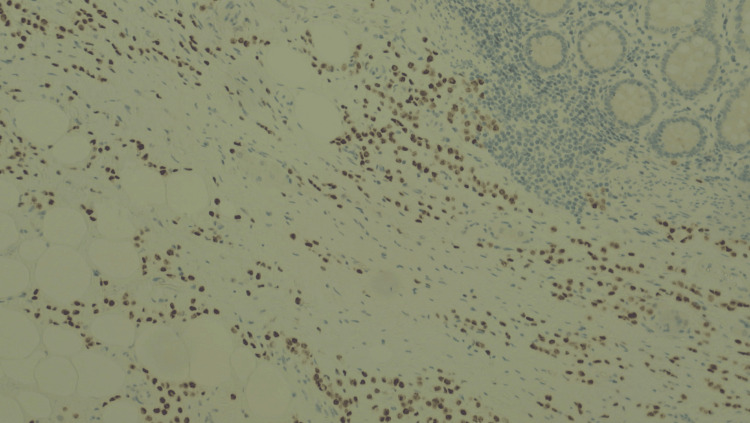
Immunostaining of the colon specimen: estrogens. Immunostaining for estrogen receptors at 10× magnification. The tumor cells are positive for estrogen receptors.

**Figure 5:**
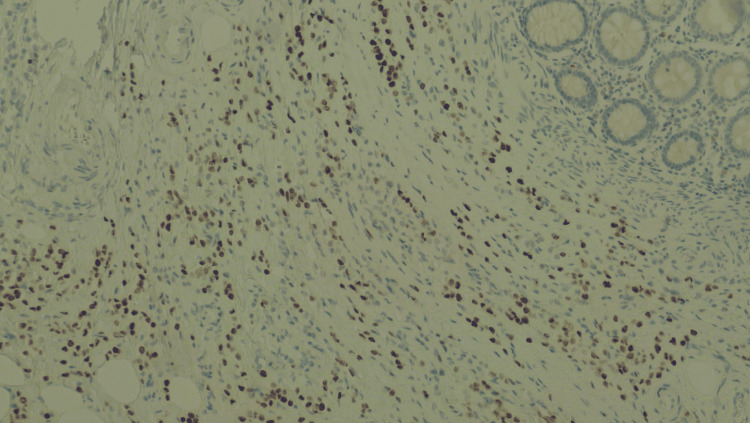
Immunostaining of the colon specimen: progesterone. Immunostaining for progesterone receptors at 10× magnification. The tumor cells are positive for progesterone receptors.

**Figure 6:**
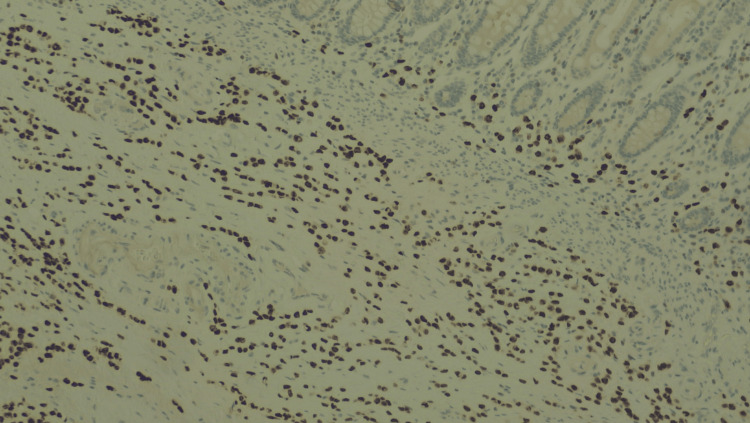
Immunostaining of the colon specimen: GATA3. Immunostaining for GATA3 at 10× magnification, positive in the tumor and negative in the colonic mucosal layer, supporting the diagnosis of metastatic breast carcinoma.

Postoperative recovery was uneventful. At final histopathology, the right hemicolectomy specimen showed a thickened and rigid colonic wall and perivisceral adipose tissue. The ileocecal valve was similarly indurated and stenotic. A 2 × 2 cm circular protrusion was noted at the base of the cecum, while the mucosa appeared macroscopically normal. Microscopic examination revealed extensive infiltration by poorly differentiated epithelial cells with relative mucosal sparing (Figure 2). Immunohistochemical analysis was performed to confirm the diagnosis and determine the tumor origin. It revealed negativity for E-cadherin, CK20, CDX2, and PAX8, and strong positivity for CK7 and GATA3 (Figures 3-6). GATA3 is a sensitive marker of luminal differentiation and is widely expressed in ILC, aiding in distinguishing breast origin from other primaries. This CK7+/CK20− profile, supported by GATA3 expression and absence of PAX8, oriented the diagnosis toward a breast origin, in line with established immunohistochemical algorithms for carcinomas of unknown primary site [20]. Hormonal profiling showed high expression of estrogen receptor (90%) and progesterone receptor (50%), HER2 negativity (score 0), and a Ki-67 proliferative index of 16%, consistent with a luminal A phenotype. Based on these findings, the diagnosis of ILC, luminal A subtype, was confirmed. All 20 retrieved lymph nodes exhibited extensive metastatic involvement.

**Figure 7:**
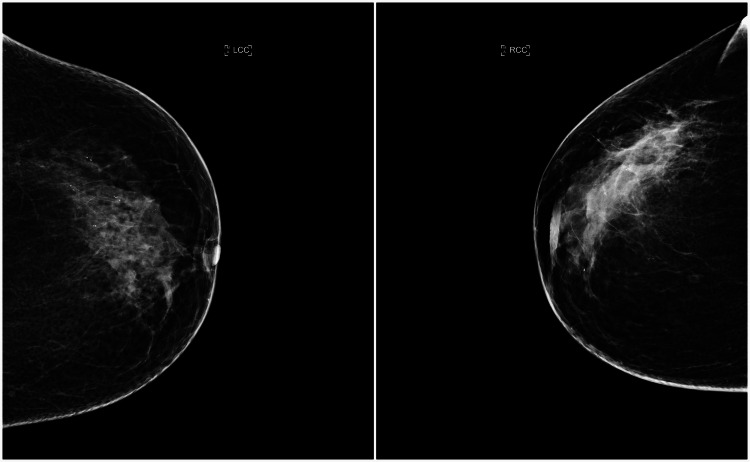
Bilateral craniocaudal (CC) mammogram. Right craniocaudal (CC) mammogram shows nipple retraction and a subtle increase in parenchymal density compared to previous imaging (not shown). These findings were considered suspicious and warranted further evaluation (BIRADS 4). Left CC mammogram is shown for comparison.

**Figure 8:**
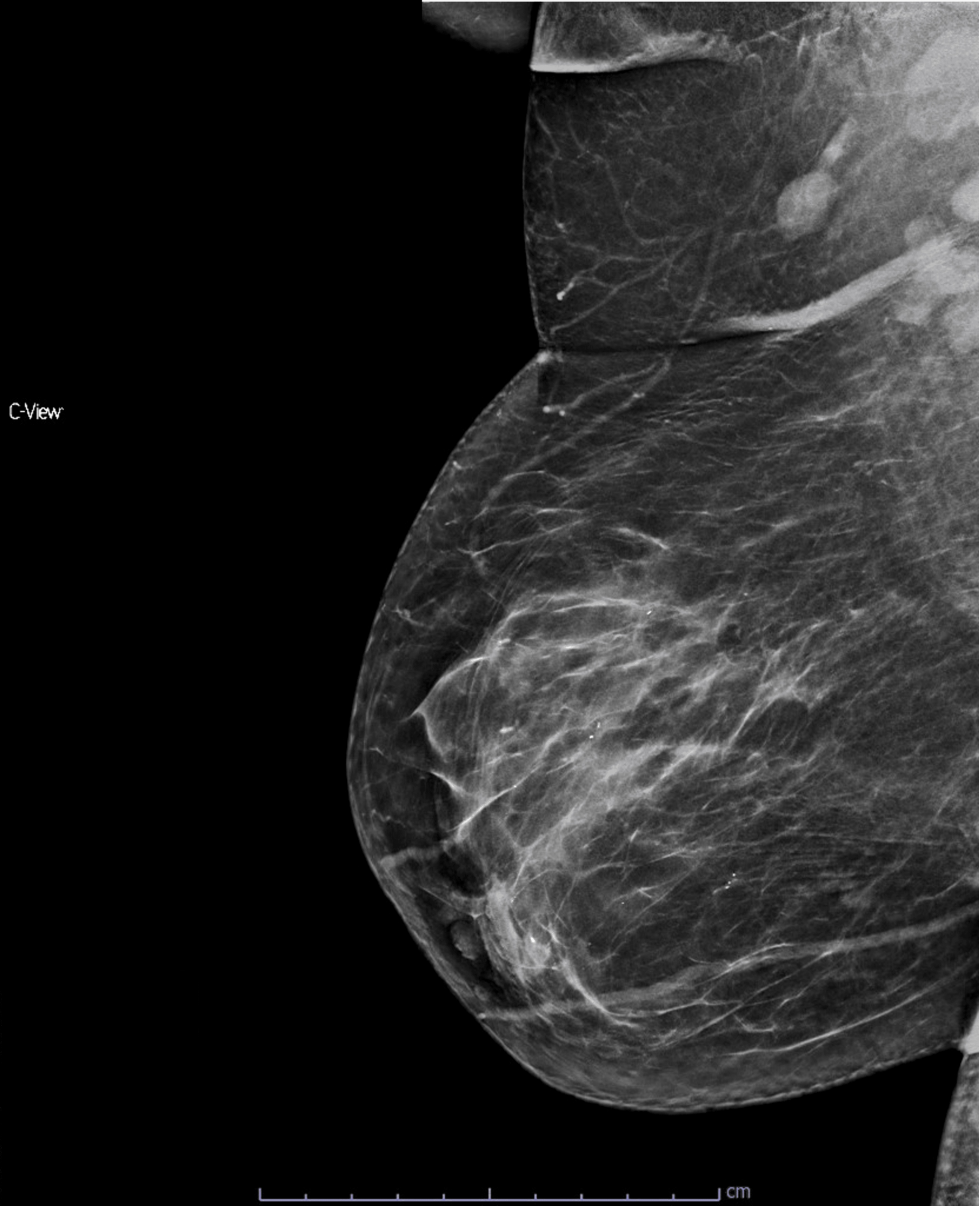
Right mediolateral oblique (MLO) mammogram. Right mediolateral oblique (MLO) mammogram shows radiologic findings classified as BIRADS 4, along with prominent right axillary lymph nodes.

When asked, the patient did not report any symptoms suggestive of breast pathology and denied noticing any changes during her routine self-examinations. Her most recent mammogram, performed nine months prior to presentation, showed stable findings compared to the previous exam, including microcalcifications in the upper outer quadrant of the left breast and a small nodular opacity in the inner quadrants of the right breast. These microcalcifications had been present in prior mammograms, consistently interpreted as dystrophic and unchanged over time. One month later, she underwent a follow-up breast ultrasound, which was unremarkable. The patient had maintained a pattern of annual mammography and ultrasound for several years, with consistently negative ultrasound results. Despite this, postoperative imaging revealed a retroareolar parenchymal density, nipple retraction, and axillary lymphadenopathy, classified as BIRADS 4 (Figures 7, 8).

**Figure 9:**
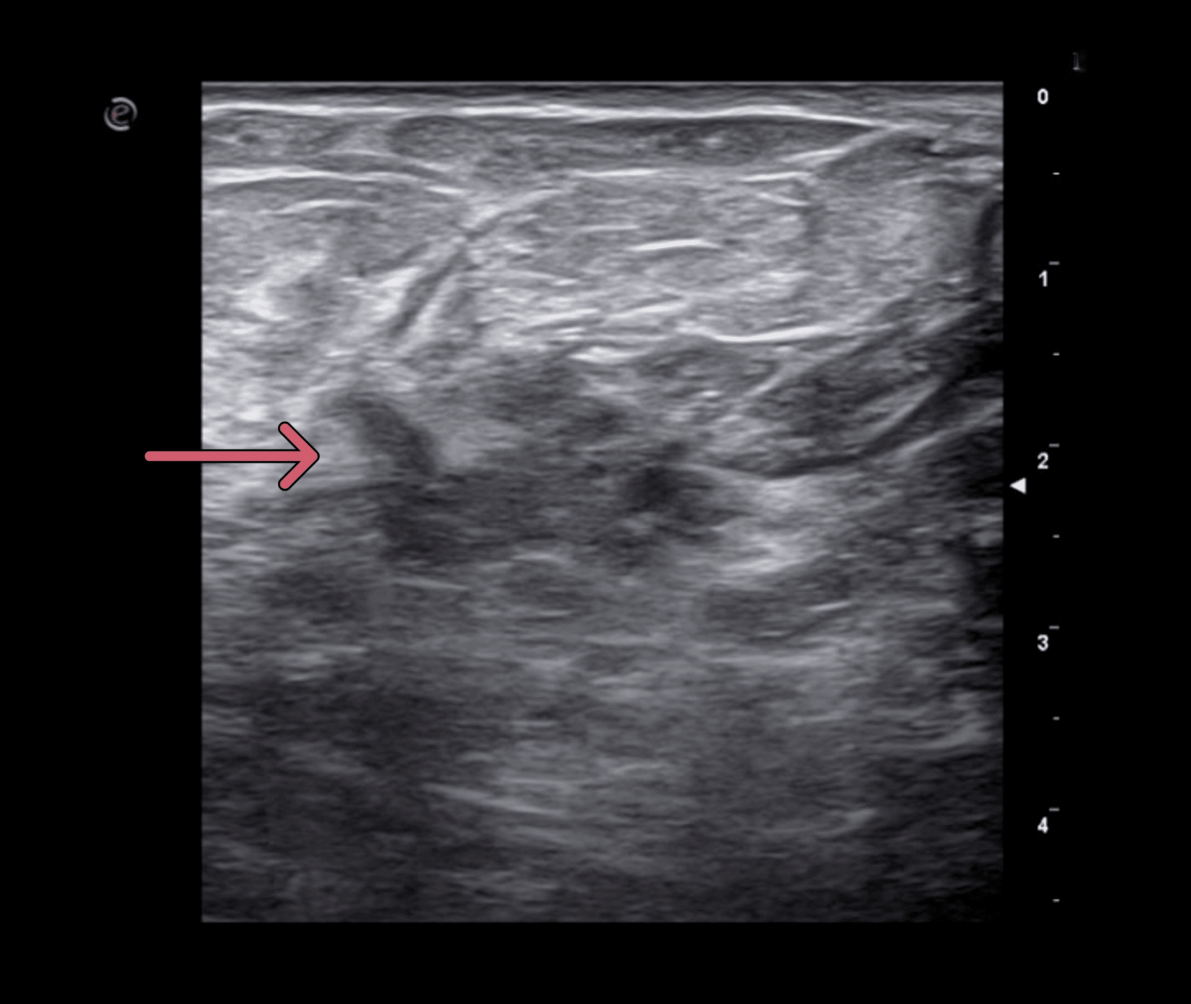
Breast ultrasound. Breast ultrasound reveals an irregular area of altered parenchymal echogenicity, with hypoechoic tissue showing infiltrative growth extending from the peri-areolar region to the outer quadrants (the arrow indicates the area of interest).

**Figure 10:**
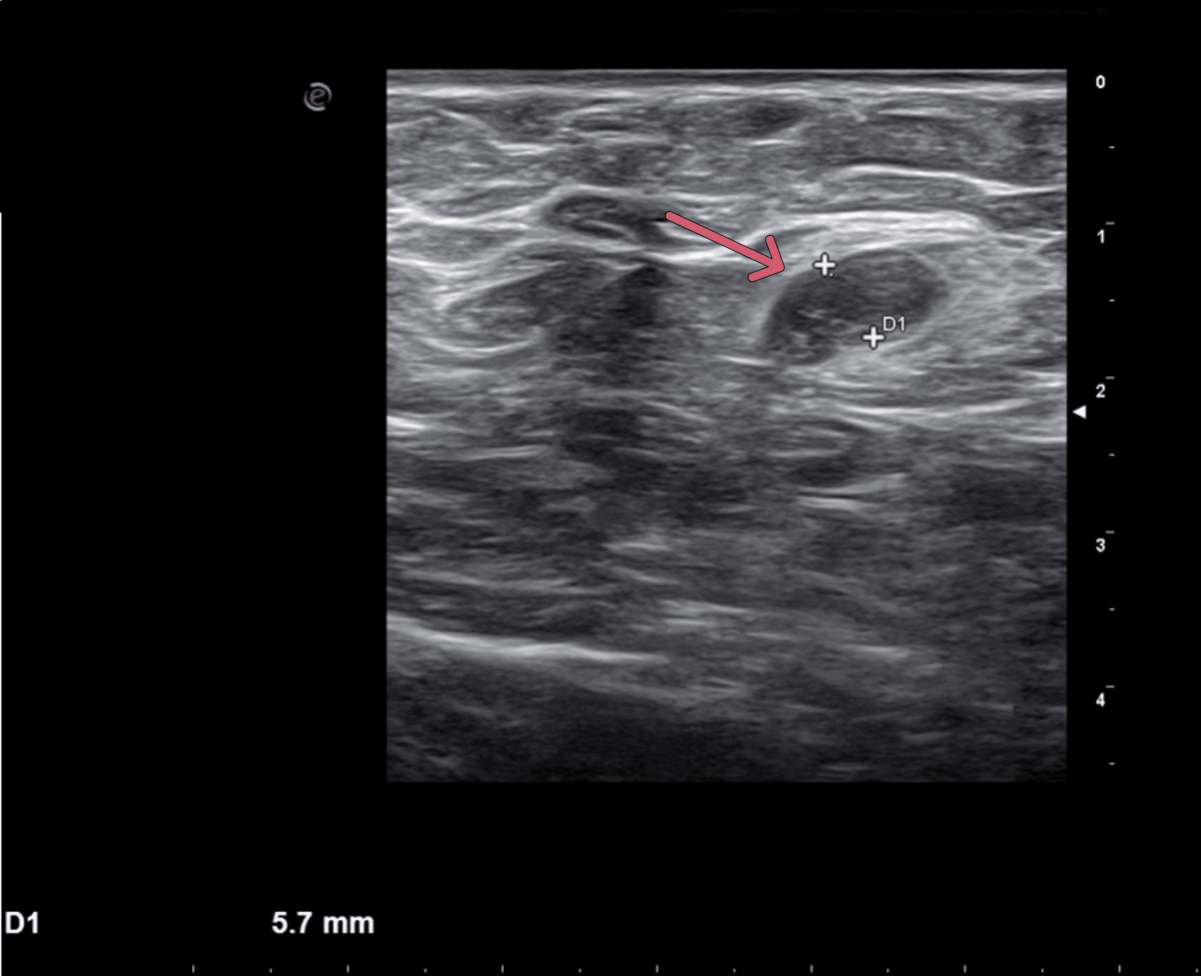
Axillary ultrasound Axillary ultrasound shows lymph nodes with suspicious features (the arrow indicates the area of interest).

**Figure 11:**
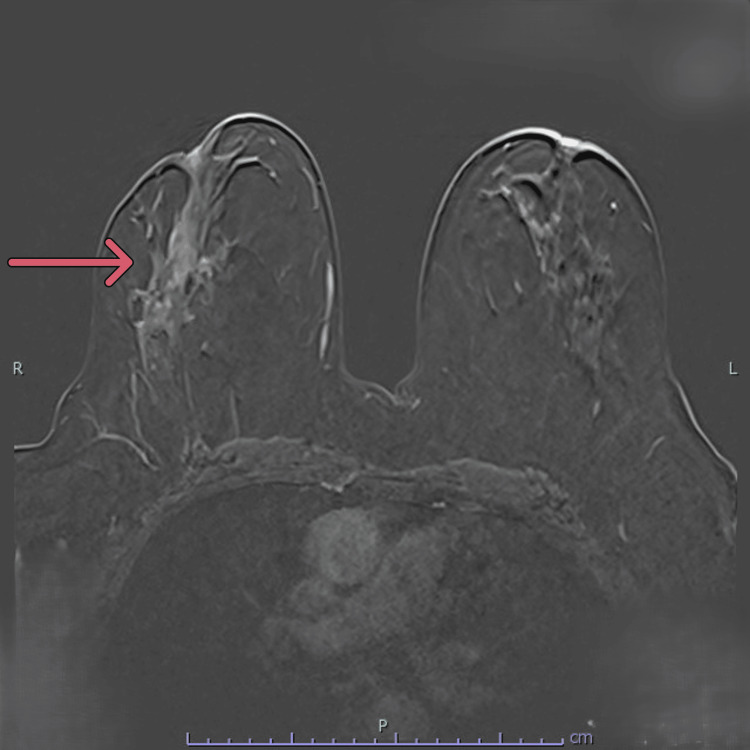
Breast magnetic resonance imaging (MRI). T1 post-contrast subtracted MRI image showing a segmental area of non-mass enhancement within normal parenchyma, highly suspicious for malignancy (the arrow indicates the area of interest).

Breast ultrasound confirmed a diffuse hypoechoic area (Figures 9, 10), while MRI showed non-mass intraparenchymal contrast enhancement extending approximately 7 cm, predominantly retroareolar (Figure 11). Minimal equivocal contrast enhancement was noted in the left breast. Total body CT ruled out distant metastatic lesions.

Following multidisciplinary review at the Breast Unit, further histologic typing was deemed unnecessary, and systemic treatment was initiated with a CDK4/6 inhibitor combined with endocrine therapy. The patient has now completed 10 cycles of ribociclib and letrozole. At one-year follow-up, she is responding well to treatment, with favorable evolution documented on contrast-enhanced CT scan. Repeat breast imaging is planned to assess the response of the primary tumor and re-evaluate the possibility of surgical intervention.

## Discussion

This case highlights an unusual and delayed diagnosis of breast cancer metastasis to the colon despite regular surveillance.

Breast cancer metastases to the gastrointestinal tract are uncommon, and colonic involvement as the first manifestation of an undetected primary tumor is extremely rare [5-18]. While breast cancer is the most frequently diagnosed cancer among women in the European Union, with over 355,000 new cases and 92,000 deaths reported in 2020 [21], gastrointestinal metastases remain a rare occurrence, with colonic involvement documented in only 3-12% of cases. Zhou et al. described a case of sigmoid metastasis diagnosed ten years after mastectomy [6], while Uygun et al. reported multiple gastrointestinal metastases in patients with known breast cancer histories [18]. Similarly, other authors documented colonic involvement years after treatment of a primary tumor [8,9,11]. By contrast, only Zaanona et al. reported a case of colonic metastasis from lobular breast cancer in a patient without prior breast malignancy [19]. In our case, the patient had no personal cancer history and underwent annual screenings, exceeding standard regional protocols in Lombardy, where women from 50 to 74 years old are usually offered a mammography every two years. This raises concerns about the adequacy of current breast cancer screening programs, as studies suggest annual mammography may improve life-years gained, although biennial screening remains preferred for efficiency and reduced false positives [22]. A recent US Preventive Services Task Force (USPSTF) study was inconclusive, highlighting the need for further research [23]. In June 2024, the USPSTF updated its recommendations to include biennial screening mammography for average-risk women aged 40 to 74, based on a commissioned systematic review and CISNET modeling. By contrast, the American College of Radiology (ACR) recommends annual screening mammography starting at age 40 for women at average risk, emphasizing that consistent yearly screening maximizes benefits and should continue as long as the individual is in good health and willing to undergo follow-up testing if needed [24]. In this case, the frequency of imaging was appropriate: the patient underwent annual mammography and ultrasound on her own initiative, and the most recent ultrasound was unremarkable. While prior mammograms showed unchanged findings over time - a common criterion for benign interpretation - the diagnosis of ILC was ultimately prompted by new clinical signs. This underscores the interpretive challenges of ILC and, despite adherence to recommended screening intervals, prompts reflection on the adequacy of current programs in detecting subtle or evolving disease.

This case also underscores the diagnostic challenges of ILC. Mammography often fails to detect ILC, particularly in its earlier stages, as hallmark features such as nipple retraction and increased density may not be apparent until later in the disease course. In our patient, these findings were present at the time of diagnosis but had not been visible on imaging performed nine months earlier. She had not noticed any breast changes, including nipple retraction, prior to diagnosis. Ultrasound can also be inconclusive, and ILC’s diffuse infiltration pattern complicates early detection [5]. Supplemental imaging, such as contrast-enhanced mammography or MRI, may improve diagnostic accuracy when conventional screening is inconclusive, though these second-level exams are not routinely offered. The American College of Radiology (ACR) recommends breast MRI for women at higher-than-average risk [23]. MRI has the highest sensitivity for ILC (93-100%), but limited specificity and frequent overestimation of lesion size can affect interpretation and surgical planning. Moreover, its impact on survival remains unproven [25]. In our case, rapid progression was suggested by the emergence of axillary adenopathy, absent one year earlier, indicating possible lymph node metastases.

Histologic diagnosis was also difficult due to ILC's growth pattern, which largely spared the mucosal layer, making endoscopic biopsy inadequate. Similar cases exist, including Tsujikawa et al.’s report of ILC presenting as colonic linitis plastica, requiring PET/CT imaging for confirmation [5].

When endoscopic biopsy fails to yield a definitive histotype, expanded diagnostics should be considered. Standard CT imaging may be insufficient, and 18F-FDG PET could help in challenging cases. Given breast cancer’s high incidence, reassessment with mammography and ultrasound may be warranted in unclear colorectal neoplasms.

Finally, this case highlights the value of multidisciplinary management in complex oncologic cases. MDT discussion can change the indication in a significant number of cases, with a demonstrable impact on the patient's prognosis [2,26]. In this particular case, although the diagnosis was made by surgery, the subsequent multidisciplinary management led to diagnostic evaluation and initiation of appropriate treatment in a short period of time.

## Conclusions

This case highlights the importance of considering metastatic breast cancer in patients presenting with non-adenocarcinoma colon lesions, even in the absence of prior breast disease or negative screening history. The diagnostic process can be complex and may require surgical excision when endoscopic biopsy proves inconclusive. Multidisciplinary collaboration plays a pivotal role in navigating such diagnostic uncertainty, ensuring accurate histopathological interpretation and appropriate management. These findings underscore the need for heightened clinical awareness and may prompt reconsideration of diagnostic pathways in atypical colorectal presentations.
